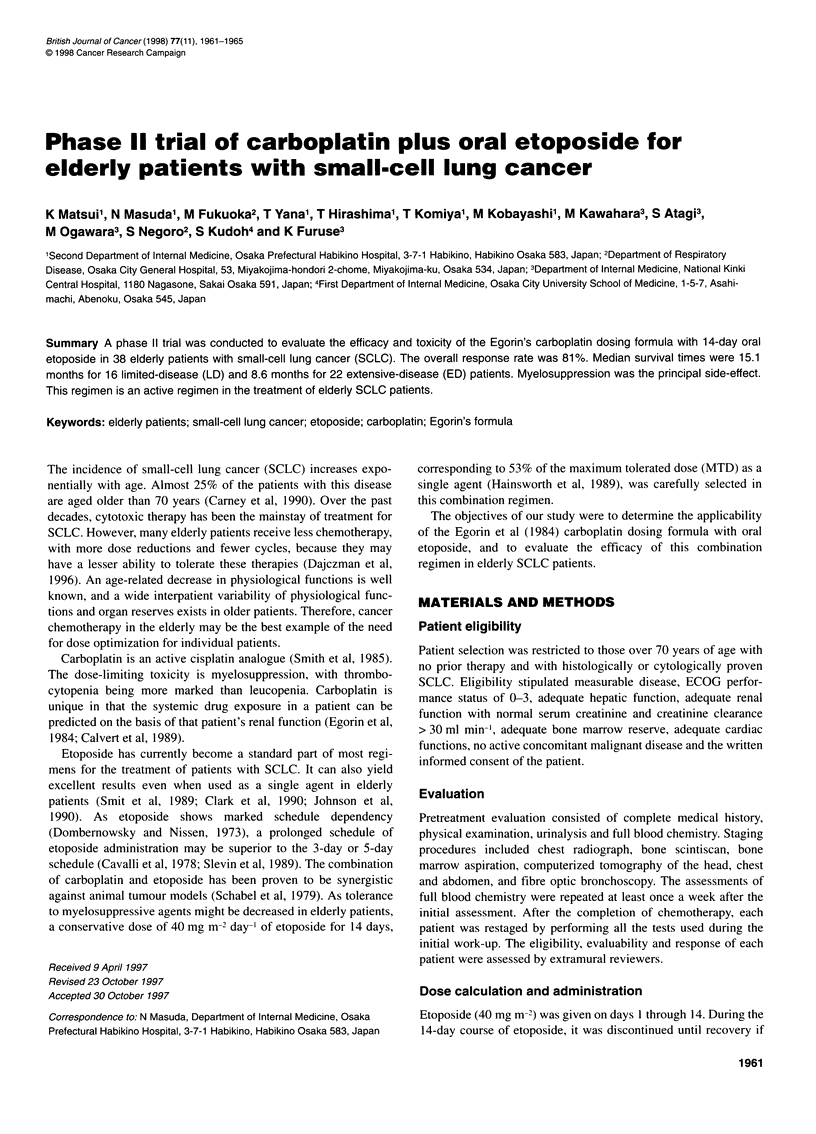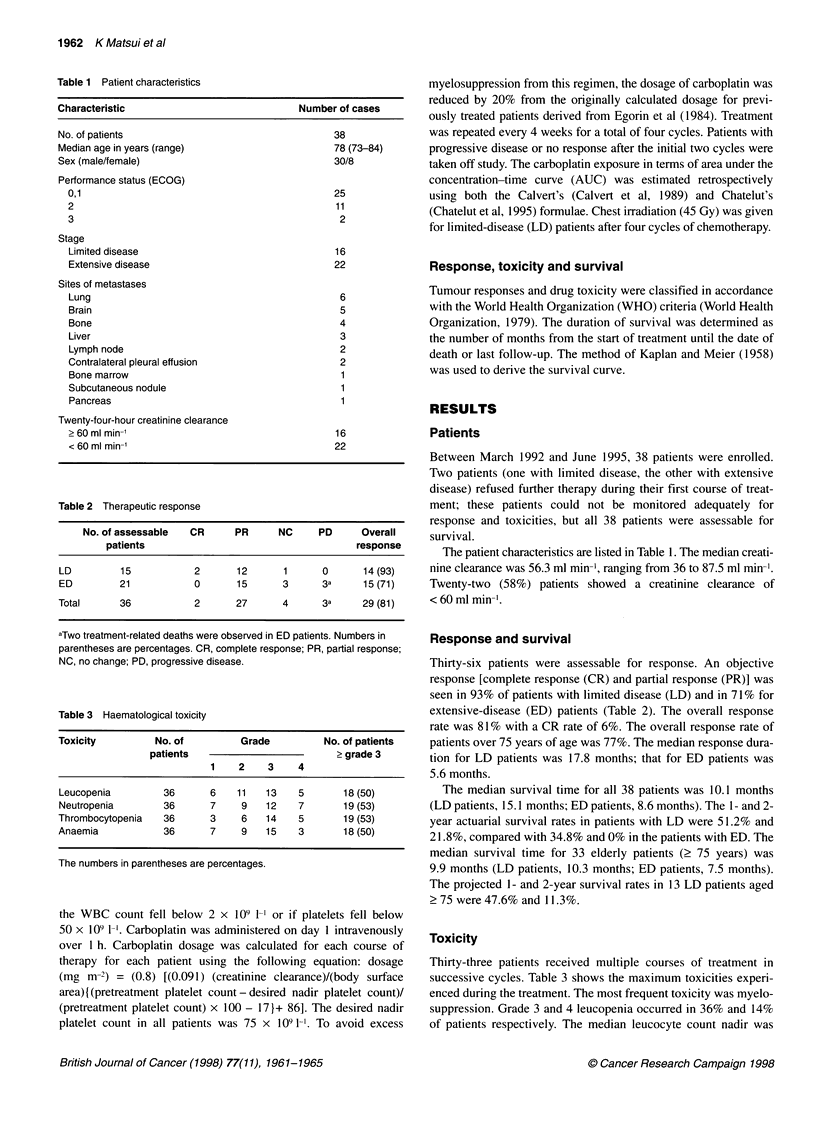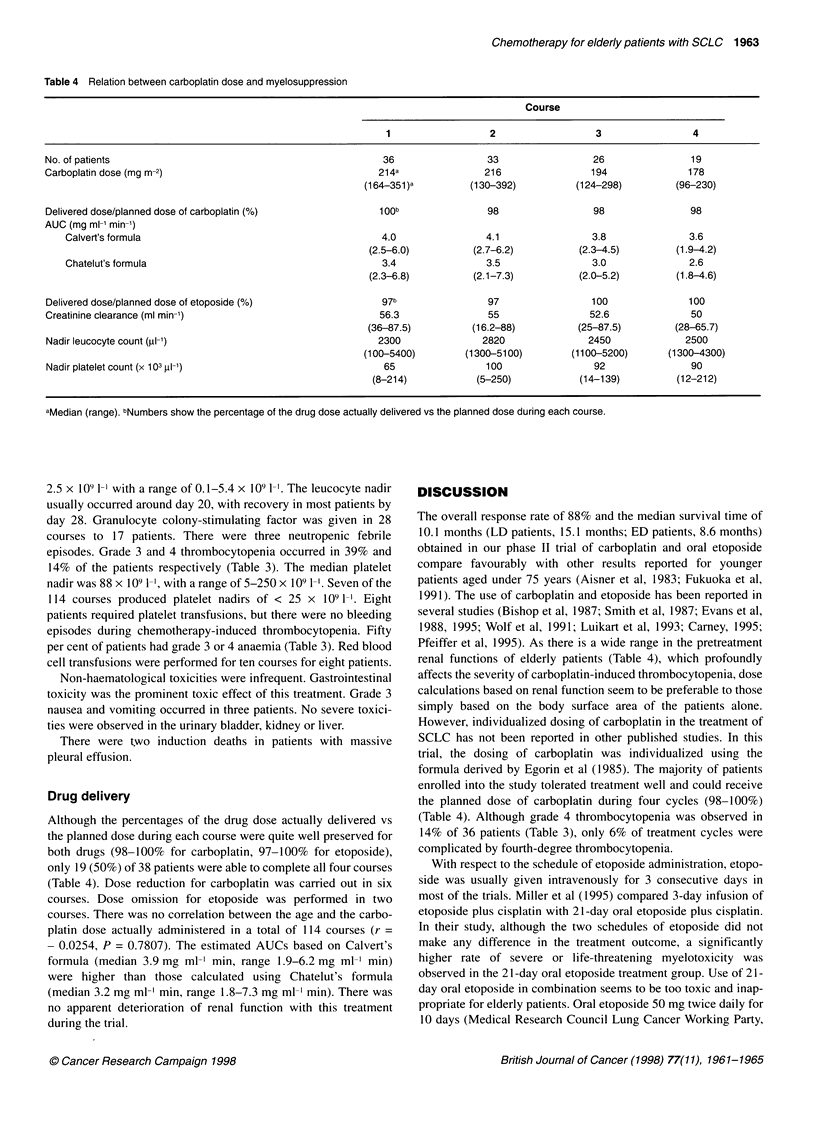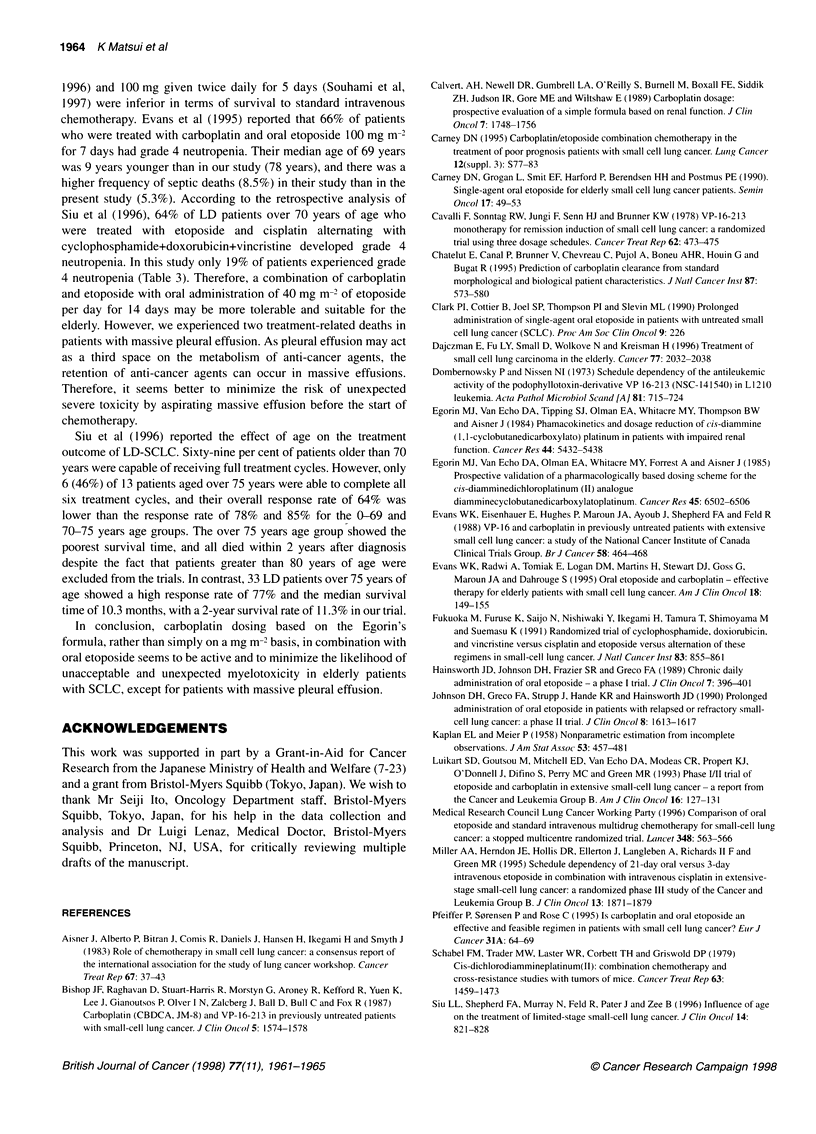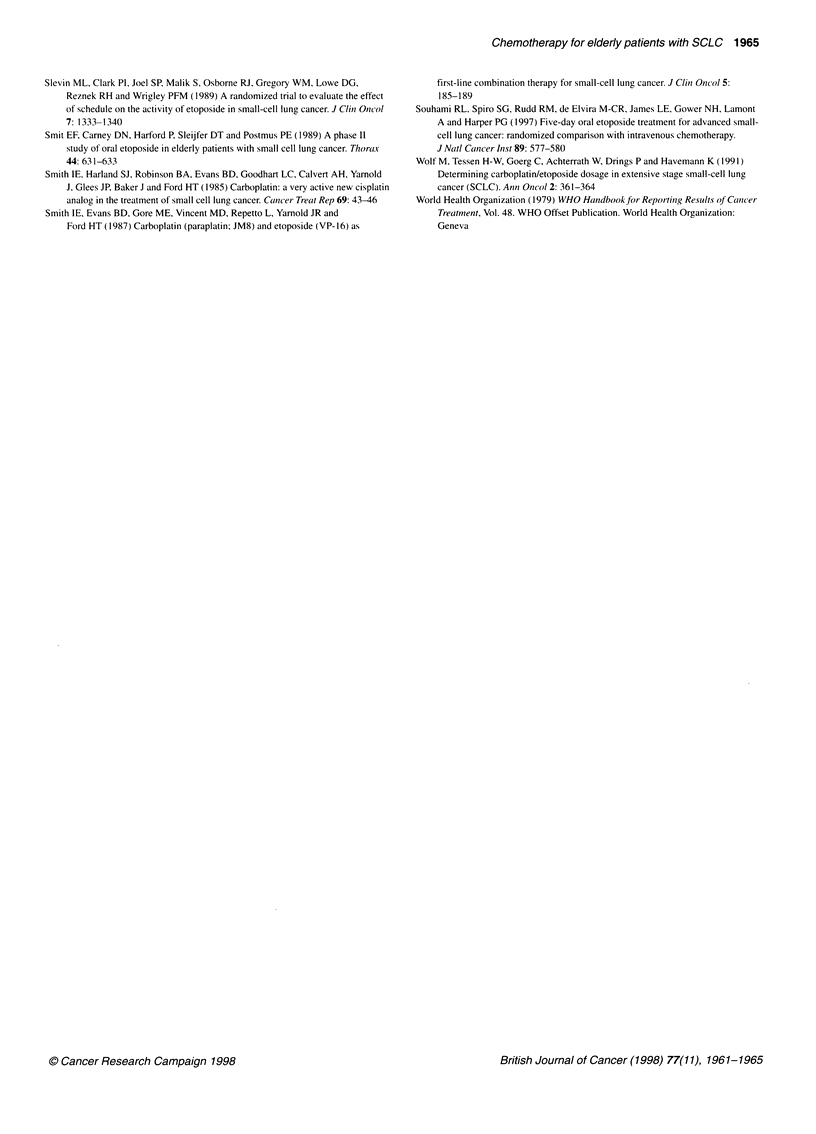# Phase II trial of carboplatin plus oral etoposide for elderly patients with small-cell lung cancer.

**DOI:** 10.1038/bjc.1998.325

**Published:** 1998-06

**Authors:** K. Matsui, N. Masuda, M. Fukuoka, T. Yana, T. Hirashima, T. Komiya, M. Kobayashi, M. Kawahara, S. Atagi, M. Ogawara, S. Negoro, S. Kudoh, K. Furuse

**Affiliations:** Second Department of Internal Medicine, Osaka Prefectural Habikino Hospital, Japan.

## Abstract

A phase II trial was conducted to evaluate the efficacy and toxicity of the Egorin's carboplatin dosing formula with 14-day oral etoposide in 38 elderly patients with small-cell lung cancer (SCLC). The overall response rate was 81%. Median survival times were 15.1 months for 16 limited-disease (LD) and 8.6 months for 22 extensive-disease (ED) patients. Myelosuppression was the principal side-effect. This regimen is an active regimen in the treatment of elderly SCLC patients.


British Journal of Cancer (1998) 77(11), 1961–1965
© 1998 Cancer Research Campaign

### Phase II trial of carboplatin plus oral etoposide for elderly patients with small-cell lung cancer

**K Matsui[1], N Masuda[1], M Fukuoka[2], T Yana[1], T Hirashima[1], T Komiya[1], M Kobayashi[1], M Kawahara[3], S Atagi[3], M Ogawara[3], S Negoro[2], S Kudoh[4] and K Furuse[3]**

[1]Second Department of Internal Medicine, Osaka Prefectural Habikino Hospital, 3-7-1 Habikino, Habikino Osaka 583, Japan; [2]Department of Respiratory Disease, Osaka City General Hospital, 53, Miyakojima-hondori 2-chome, Miyakojima-ku, Osaka 534, Japan; [3]Department of Internal Medicine, National Kinki Central Hospital, 1180 Nagasone, Sakai Osaka 591, Japan; [4]First Department of Internal Medicine, Osaka City University School of Medicine, 1-5-7, Asahi-machi, Abenoku, Osaka 545, Japan

**Summary** A phase II trial was conducted to evaluate the efficacy and toxicity of the Egorin's carboplatin dosing formula with 14-day oral etoposide in 38 elderly patients with small-cell lung cancer (SCLC). The overall response rate was 81%. Median survival times were 15.1 months for 16 limited-disease (LD) and 8.6 months for 22 extensive-disease (ED) patients. Myelosuppression was the principal side-effect. This regimen is an active regimen in the treatment of elderly SCLC patients.

**Keywords:** elderly patients; small-cell lung cancer; etoposide; carboplatin; Egorin's formula

The incidence of small-cell lung cancer (SCLC) increases exponentially with age. Almost 25% of the patients with this disease are aged older than 70 years (Carney et al, 1990). Over the past decades, cytotoxic therapy has been the mainstay of treatment for SCLC. However, many elderly patients receive less chemotherapy, with more dose reductions and fewer cycles, because they may have a lesser ability to tolerate these therapies (Dajczman et al, 1996). An age-related decrease in physiological functions is well known, and a wide interpatient variability of physiological functions and organ reserves exists in older patients. Therefore, cancer chemotherapy in the elderly may be the best example of the need for dose optimization for individual patients.

Carboplatin is an active cisplatin analogue (Smith et al, 1985). The dose-limiting toxicity is myelosuppression, with thrombocytopenia being more marked than leucopenia. Carboplatin is unique in that the systemic drug exposure in a patient can be predicted on the basis of that patient's renal function (Egorin et al, 1984; Calvert et al, 1989).

Etoposide has currently become a standard part of most regimens for the treatment of patients with SCLC. It can also yield excellent results even when used as a single agent in elderly patients (Smit et al, 1989; Clark et al, 1990; Johnson et al, 1990). As etoposide shows marked schedule dependency (Dombernowsky and Nissen, 1973), a prolonged schedule of etoposide administration may be superior to the 3-day or 5-day schedule (Cavalli et al, 1978; Slevin et al, 1989). The combination of carboplatin and etoposide has been proven to be synergistic against animal tumour models (Schabel et al, 1979). As tolerance to myelosuppressive agents might be decreased in elderly patients, a conservative dose of 40 mg $m^{-2}$ $day^{-1}$ of etoposide for 14 days, corresponding to 53% of the maximum tolerated dose (MTD) as a single agent (Hainsworth et al, 1989), was carefully selected in this combination regimen.

The objectives of our study were to determine the applicability of the Egorin et al (1984) carboplatin dosing formula with oral etoposide, and to evaluate the efficacy of this combination regimen in elderly SCLC patients.

*Received 9 April 1997*
*Revised 23 October 1997*
*Accepted 30 October 1997*

*Correspondence to:* N Masuda, Department of Internal Medicine, Osaka Prefectural Habikino Hospital, 3-7-1 Habikino, Habikino Osaka 583, Japan

### MATERIALS AND METHODS

### Patient eligibility

Patient selection was restricted to those over 70 years of age with no prior therapy and with histologically or cytologically proven SCLC. Eligibility stipulated measurable disease, ECOG performance status of 0–3, adequate hepatic function, adequate renal function with normal serum creatinine and creatinine clearance > 30 ml $min^{-1}$, adequate bone marrow reserve, adequate cardiac functions, no active concomitant malignant disease and the written informed consent of the patient.

### Evaluation

Pretreatment evaluation consisted of complete medical history, physical examination, urinalysis and full blood chemistry. Staging procedures included chest radiograph, bone scintiscan, bone marrow aspiration, computerized tomography of the head, chest and abdomen, and fibre optic bronchoscopy. The assessments of full blood chemistry were repeated at least once a week after the initial assessment. After the completion of chemotherapy, each patient was restaged by performing all the tests used during the initial work-up. The eligibility, evaluability and response of each patient were assessed by extramural reviewers.

### Dose calculation and administration

Etoposide (40 mg $m^{-2}$) was given on days 1 through 14. During the 14-day course of etoposide, it was discontinued until recovery if

**Table 1** Patient characteristics

| Characteristic | Number of cases |
|---|---|
| No. of patients | 38 |
| Median age in years (range) | 78 (73–84) |
| Sex (male/female) | 30/8 |
| Performance status (ECOG) | |
| 0,1 | 25 |
| 2 | 11 |
| 3 | 2 |
| Stage | |
| Limited disease | 16 |
| Extensive disease | 22 |
| Sites of metastases | |
| Lung | 6 |
| Brain | 5 |
| Bone | 4 |
| Liver | 3 |
| Lymph node | 2 |
| Contralateral pleural effusion | 2 |
| Bone marrow | 1 |
| Subcutaneous nodule | 1 |
| Pancreas | 1 |
| Twenty-four-hour creatinine clearance | |
| ≥ 60 ml $min^{-1}$ | 16 |
| < 60 ml $min^{-1}$ | 22 |

**Table 2** Therapeutic response

| | No. of assessable patients | CR | PR | NC | PD | Overall response |
|---|---|---|---|---|---|---|
| LD | 15 | 2 | 12 | 1 | 0 | 14 (93) |
| ED | 21 | 0 | 15 | 3 | 3[a] | 15 (71) |
| Total | 36 | 2 | 27 | 4 | 3[a] | 29 (81) |

[a]Two treatment-related deaths were observed in ED patients. Numbers in parentheses are percentages. CR, complete response; PR, partial response; NC, no change; PD, progressive disease.

**Table 3** Haematological toxicity

| Toxicity | No. of patients | Grade | | | | No. of patients ≥ grade 3 |
|---|---|---|---|---|---|---|
| | | 1 | 2 | 3 | 4 | |
| Leucopenia | 36 | 6 | 11 | 13 | 5 | 18 (50) |
| Neutropenia | 36 | 7 | 9 | 12 | 7 | 19 (53) |
| Thrombocytopenia | 36 | 3 | 6 | 14 | 5 | 19 (53) |
| Anaemia | 36 | 7 | 9 | 15 | 3 | 18 (50) |

The numbers in parentheses are percentages.

the WBC count fell below $2 \times 10^9\ l^{-1}$ or if platelets fell below $50 \times 10^9\ l^{-1}$. Carboplatin was administered on day 1 intravenously over 1 h. Carboplatin dosage was calculated for each course of therapy for each patient using the following equation: dosage (mg $m^{-2}$) = (0.8) [(0.091) (creatinine clearance)/(body surface area){(pretreatment platelet count – desired nadir platelet count)/(pretreatment platelet count) × 100 – 17}+ 86]. The desired nadir platelet count in all patients was $75 \times 10^9\ l^{-1}$. To avoid excess myelosuppression from this regimen, the dosage of carboplatin was reduced by 20% from the originally calculated dosage for previously treated patients derived from Egorin et al (1984). Treatment was repeated every 4 weeks for a total of four cycles. Patients with progressive disease or no response after the initial two cycles were taken off study. The carboplatin exposure in terms of area under the concentration–time curve (AUC) was estimated retrospectively using both the Calvert's (Calvert et al, 1989) and Chatelut's (Chatelut et al, 1995) formulae. Chest irradiation (45 Gy) was given for limited-disease (LD) patients after four cycles of chemotherapy.

### Response, toxicity and survival

Tumour responses and drug toxicity were classified in accordance with the World Health Organization (WHO) criteria (World Health Organization, 1979). The duration of survival was determined as the number of months from the start of treatment until the date of death or last follow-up. The method of Kaplan and Meier (1958) was used to derive the survival curve.

### RESULTS

### Patients

Between March 1992 and June 1995, 38 patients were enrolled. Two patients (one with limited disease, the other with extensive disease) refused further therapy during their first course of treatment; these patients could not be monitored adequately for response and toxicities, but all 38 patients were assessable for survival.

The patient characteristics are listed in Table 1. The median creatinine clearance was 56.3 ml $min^{-1}$, ranging from 36 to 87.5 ml $min^{-1}$. Twenty-two (58%) patients showed a creatinine clearance of < 60 ml $min^{-1}$.

### Response and survival

Thirty-six patients were assessable for response. An objective response [complete response (CR) and partial response (PR)] was seen in 93% of patients with limited disease (LD) and in 71% for extensive-disease (ED) patients (Table 2). The overall response rate was 81% with a CR rate of 6%. The overall response rate of patients over 75 years of age was 77%. The median response duration for LD patients was 17.8 months; that for ED patients was 5.6 months.

The median survival time for all 38 patients was 10.1 months (LD patients, 15.1 months; ED patients, 8.6 months). The 1- and 2-year actuarial survival rates in patients with LD were 51.2% and 21.8%, compared with 34.8% and 0% in the patients with ED. The median survival time for 33 elderly patients (≥ 75 years) was 9.9 months (LD patients, 10.3 months; ED patients, 7.5 months). The projected 1- and 2-year survival rates in 13 LD patients aged ≥ 75 were 47.6% and 11.3%.

### Toxicity

Thirty-three patients received multiple courses of treatment in successive cycles. Table 3 shows the maximum toxicities experienced during the treatment. The most frequent toxicity was myelosuppression. Grade 3 and 4 leucopenia occurred in 36% and 14% of patients respectively. The median leucocyte count nadir was

 © Cancer Research Campaign 1998

**Table 4** Relation between carboplatin dose and myelosuppression

| | Course | | | |
|---|---|---|---|---|
| | 1 | 2 | 3 | 4 |
| No. of patients | 36 | 33 | 26 | 19 |
| Carboplatin dose (mg $m^{-2}$) | 214[a] | 216 | 194 | 178 |
| | (164–351)[a] | (130–392) | (124–298) | (96–230) |
| Delivered dose/planned dose of carboplatin (%) | 100[b] | 98 | 98 | 98 |
| AUC (mg $ml^{-1}$ $min^{-1}$) | | | | |
| Calvert's formula | 4.0 | 4.1 | 3.8 | 3.6 |
| | (2.5–6.0) | (2.7–6.2) | (2.3–4.5) | (1.9–4.2) |
| Chatelut's formula | 3.4 | 3.5 | 3.0 | 2.6 |
| | (2.3–6.8) | (2.1–7.3) | (2.0–5.2) | (1.8–4.6) |
| Delivered dose/planned dose of etoposide (%) | 97[b] | 97 | 100 | 100 |
| Creatinine clearance (ml $min^{-1}$) | 56.3 | 55 | 52.6 | 50 |
| | (36–87.5) | (16.2–88) | (25–87.5) | (28–65.7) |
| Nadir leucocyte count ($\mu l^{-1}$) | 2300 | 2820 | 2450 | 2500 |
| | (100–5400) | (1300–5100) | (1100–5200) | (1300–4300) |
| Nadir platelet count ($\times 10^3$ $\mu l^{-1}$) | 65 | 100 | 92 | 90 |
| | (8–214) | (5–250) | (14–139) | (12–212) |

[a]Median (range). [b]Numbers show the percentage of the drug dose actually delivered vs the planned dose during each course.

$2.5 \times 10^9$ $l^{-1}$ with a range of 0.1–5.4 $\times 10^9$ $l^{-1}$. The leucocyte nadir usually occurred around day 20, with recovery in most patients by day 28. Granulocyte colony-stimulating factor was given in 28 courses to 17 patients. There were three neutropenic febrile episodes. Grade 3 and 4 thrombocytopenia occurred in 39% and 14% of the patients respectively (Table 3). The median platelet nadir was $88 \times 10^9$ $l^{-1}$, with a range of 5–250 $\times 10^9$ $l^{-1}$. Seven of the 114 courses produced platelet nadirs of $< 25 \times 10^9$ $l^{-1}$. Eight patients required platelet transfusions, but there were no bleeding episodes during chemotherapy-induced thrombocytopenia. Fifty per cent of patients had grade 3 or 4 anaemia (Table 3). Red blood cell transfusions were performed for ten courses for eight patients.

Non-haematological toxicities were infrequent. Gastrointestinal toxicity was the prominent toxic effect of this treatment. Grade 3 nausea and vomiting occurred in three patients. No severe toxicities were observed in the urinary bladder, kidney or liver.

There were two induction deaths in patients with massive pleural effusion.

### Drug delivery

Although the percentages of the drug dose actually delivered vs the planned dose during each course were quite well preserved for both drugs (98–100% for carboplatin, 97–100% for etoposide), only 19 (50%) of 38 patients were able to complete all four courses (Table 4). Dose reduction for carboplatin was carried out in six courses. Dose omission for etoposide was performed in two courses. There was no correlation between the age and the carboplatin dose actually administered in a total of 114 courses ($r = -0.0254$, $P = 0.7807$). The estimated AUCs based on Calvert's formula (median 3.9 mg $ml^{-1}$ min, range 1.9–6.2 mg $ml^{-1}$ min) were higher than those calculated using Chatelut's formula (median 3.2 mg $ml^{-1}$ min, range 1.8–7.3 mg $ml^{-1}$ min). There was no apparent deterioration of renal function with this treatment during the trial.

### DISCUSSION

The overall response rate of 88% and the median survival time of 10.1 months (LD patients, 15.1 months; ED patients, 8.6 months) obtained in our phase II trial of carboplatin and oral etoposide compare favourably with other results reported for younger patients aged under 75 years (Aisner et al, 1983; Fukuoka et al, 1991). The use of carboplatin and etoposide has been reported in several studies (Bishop et al, 1987; Smith et al, 1987; Evans et al, 1988, 1995; Wolf et al, 1991; Luikart et al, 1993; Carney, 1995; Pfeiffer et al, 1995). As there is a wide range in the pretreatment renal functions of elderly patients (Table 4), which profoundly affects the severity of carboplatin-induced thrombocytopenia, dose calculations based on renal function seem to be preferable to those simply based on the body surface area of the patients alone. However, individualized dosing of carboplatin in the treatment of SCLC has not been reported in other published studies. In this trial, the dosing of carboplatin was individualized using the formula derived by Egorin et al (1985). The majority of patients enrolled into the study tolerated treatment well and could receive the planned dose of carboplatin during four cycles (98–100%) (Table 4). Although grade 4 thrombocytopenia was observed in 14% of 36 patients (Table 3), only 6% of treatment cycles were complicated by fourth-degree thrombocytopenia.

With respect to the schedule of etoposide administration, etoposide was usually given intravenously for 3 consecutive days in most of the trials. Miller et al (1995) compared 3-day infusion of etoposide plus cisplatin with 21-day oral etoposide plus cisplatin. In their study, although the two schedules of etoposide did not make any difference in the treatment outcome, a significantly higher rate of severe or life-threatening myelotoxicity was observed in the 21-day oral etoposide treatment group. Use of 21-day oral etoposide in combination seems to be too toxic and inappropriate for elderly patients. Oral etoposide 50 mg twice daily for 10 days (Medical Research Council Lung Cancer Working Party,

© Cancer Research Campaign 1998

1996) and 100 mg given twice daily for 5 days (Souhami et al, 1997) were inferior in terms of survival to standard intravenous chemotherapy. Evans et al (1995) reported that 66% of patients who were treated with carboplatin and oral etoposide 100 mg $m^{-2}$ for 7 days had grade 4 neutropenia. Their median age of 69 years was 9 years younger than in our study (78 years), and there was a higher frequency of septic deaths (8.5%) in their study than in the present study (5.3%). According to the retrospective analysis of Siu et al (1996), 64% of LD patients over 70 years of age who were treated with etoposide and cisplatin alternating with cyclophosphamide+doxorubicin+vincristine developed grade 4 neutropenia. In this study only 19% of patients experienced grade 4 neutropenia (Table 3). Therefore, a combination of carboplatin and etoposide with oral administration of 40 mg $m^{-2}$ of etoposide per day for 14 days may be more tolerable and suitable for the elderly. However, we experienced two treatment-related deaths in patients with massive pleural effusion. As pleural effusion may act as a third space on the metabolism of anti-cancer agents, the retention of anti-cancer agents can occur in massive effusions. Therefore, it seems better to minimize the risk of unexpected severe toxicity by aspirating massive effusion before the start of chemotherapy.

Siu et al (1996) reported the effect of age on the treatment outcome of LD-SCLC. Sixty-nine per cent of patients older than 70 years were capable of receiving full treatment cycles. However, only 6 (46%) of 13 patients aged over 75 years were able to complete all six treatment cycles, and their overall response rate of 64% was lower than the response rate of 78% and 85% for the 0–69 and 70–75 years age groups. The over 75 years age group showed the poorest survival time, and all died within 2 years after diagnosis despite the fact that patients greater than 80 years of age were excluded from the trials. In contrast, 33 LD patients over 75 years of age showed a high response rate of 77% and the median survival time of 10.3 months, with a 2-year survival rate of 11.3% in our trial.

In conclusion, carboplatin dosing based on the Egorin's formula, rather than simply on a mg $m^{-2}$ basis, in combination with oral etoposide seems to be active and to minimize the likelihood of unacceptable and unexpected myelotoxicity in elderly patients with SCLC, except for patients with massive pleural effusion.

### ACKNOWLEDGEMENTS

This work was supported in part by a Grant-in-Aid for Cancer Research from the Japanese Ministry of Health and Welfare (7-23) and a grant from Bristol-Myers Squibb (Tokyo, Japan). We wish to thank Mr Seiji Ito, Oncology Department staff, Bristol-Myers Squibb, Tokyo, Japan, for his help in the data collection and analysis and Dr Luigi Lenaz, Medical Doctor, Bristol-Myers Squibb, Princeton, NJ, USA, for critically reviewing multiple drafts of the manuscript.

### REFERENCES

Aisner J, Alberto P, Bitran J, Comis R, Daniels J, Hansen H, Ikegami H and Smyth J (1983) Role of chemotherapy in small cell lung cancer: a consensus report of the international association for the study of lung cancer workshop. *Cancer Treat Rep* **67**: 37–43

Bishop JF, Raghavan D, Stuart-Harris R, Morstyn G, Aroney R, Kefford R, Yuen K, Lee J, Gianoutsos P, Olver I N, Zalcberg J, Ball D, Bull C and Fox R (1987) Carboplatin (CBDCA, JM-8) and VP-16-213 in previously untreated patients with small-cell lung cancer. *J Clin Oncol* **5**: 1574–1578

Calvert, AH, Newell DR, Gumbrell LA, O'Reilly S, Burnell M, Boxall FE, Siddik ZH, Judson IR, Gore ME and Wiltshaw E (1989) Carboplatin dosage: prospective evaluation of a simple formula based on renal function. *J Clin Oncol* **7**: 1748–1756

Carney DN (1995) Carboplatin/etoposide combination chemotherapy in the treatment of poor prognosis patients with small cell lung cancer. *Lung Cancer* **12**(suppl. 3): S77–83

Carney DN, Grogan L, Smit EF, Harford P, Berendsen HH and Postmus PE (1990). Single-agent oral etoposide for elderly small cell lung cancer patients. *Semin Oncol* **17**: 49–53

Cavalli F, Sonntag RW, Jungi F, Senn HJ and Brunner KW (1978) VP-16-213 monotherapy for remission induction of small cell lung cancer: a randomized trial using three dosage schedules. *Cancer Treat Rep* **62**: 473–475

Chatelut E, Canal P, Brunner V, Chevreau C, Pujol A, Boneu AHR, Houin G and Bugat R (1995) Prediction of carboplatin clearance from standard morphological and biological patient characteristics. *J Natl Cancer Inst* **87**: 573–580

Clark PI, Cottier B, Joel SP, Thompson PI and Slevin ML (1990) Prolonged administration of single-agent oral etoposide in patients with untreated small cell lung cancer (SCLC). *Proc Am Soc Clin Oncol* **9**: 226

Dajczman E, Fu LY, Small D, Wolkove N and Kreisman H (1996) Treatment of small cell lung carcinoma in the elderly. *Cancer* **77**: 2032–2038

Dombernowsky P and Nissen NI (1973) Schedule dependency of the antileukemic activity of the podophyllotoxin-derivative VP 16-213 (NSC-141540) in L1210 leukemia. *Acta Pathol Microbiol Scand [A]* **81**: 715–724

Egorin MJ, Van Echo DA, Tipping SJ, Olman EA, Whitacre MY, Thompson BW and Aisner J (1984) Phamacokinetics and dosage reduction of *cis*-diammine (1,1-cyclobutanedicarboxylato) platinum in patients with impaired renal function. *Cancer Res* **44**: 5432–5438

Egorin MJ, Van Echo DA, Olman EA, Whitacre MY, Forrest A and Aisner J (1985) Prospective validation of a pharmacologically based dosing scheme for the *cis*-diamminedichloroplatinum (II) analogue diamminecyclobutanedicarboxylatoplatinum. *Cancer Res* **45**: 6502–6506

Evans WK, Eisenhauer E, Hughes P, Maroun JA, Ayoub J, Shepherd FA and Feld R (1988) VP-16 and carboplatin in previously untreated patients with extensive small cell lung cancer: a study of the National Cancer Institute of Canada Clinical Trials Group. *Br J Cancer* **58**: 464–468

Evans WK, Radwi A, Tomiak E, Logan DM, Martins H, Stewart DJ, Goss G, Maroun JA and Dahrouge S (1995) Oral etoposide and carboplatin – effective therapy for elderly patients with small cell lung cancer. *Am J Clin Oncol* **18**: 149–155

Fukuoka M, Furuse K, Saijo N, Nishiwaki Y, Ikegami H, Tamura T, Shimoyama M and Suemasu K (1991) Randomized trial of cyclophosphamide, doxiorubicin, and vincristine versus cisplatin and etoposide versus alternation of these regimens in small-cell lung cancer. *J Natl Cancer Inst* **83**: 855–861

Hainsworth JD, Johnson DH, Frazier SR and Greco FA (1989) Chronic daily administration of oral etoposide – a phase I trial. *J Clin Oncol* **7**: 396–401

Johnson DH, Greco FA, Strupp J, Hande KR and Hainsworth JD (1990) Prolonged administration of oral etoposide in patients with relapsed or refractory small-cell lung cancer: a phase II trial. *J Clin Oncol* **8**: 1613–1617

Kaplan EL and Meier P (1958) Nonparametric estimation from incomplete observations. *J Am Stat Assoc* **53**: 457–481

Luikart SD, Goutsou M, Mitchell ED, Van Echo DA, Modeas CR, Propert KJ, O'Donnell J, Difino S, Perry MC and Green MR (1993) Phase I/II trial of etoposide and carboplatin in extensive small-cell lung cancer – a report from the Cancer and Leukemia Group B. *Am J Clin Oncol* **16**: 127–131

Medical Research Council Lung Cancer Working Party (1996) Comparison of oral etoposide and standard intravenous multidrug chemotherapy for small-cell lung cancer: a stopped multicentre randomized trial. *Lancet* **348**: 563–566

Miller AA, Herndon JE, Hollis DR, Ellerton J, Langleben A, Richards II F and Green MR (1995) Schedule dependency of 21-day oral versus 3-day intravenous etoposide in combination with intravenous cisplatin in extensive-stage small-cell lung cancer: a randomized phase III study of the Cancer and Leukemia Group B. *J Clin Oncol* **13**: 1871–1879

Pfeiffer P, Sørensen P and Rose C (1995) Is carboplatin and oral etoposide an effective and feasible regimen in patients with small cell lung cancer? *Eur J Cancer* **31A**: 64–69

Schabel FM, Trader MW, Laster WR, Corbett TH and Griswold DP (1979) Cis-dichlorodiammineplatinum(II): combination chemotherapy and cross-resistance studies with tumors of mice. *Cancer Treat Rep* **63**: 1459–1473

Siu LL, Shepherd FA, Murray N, Feld R, Pater J and Zee B (1996) Influence of age on the treatment of limited-stage small-cell lung cancer. *J Clin Oncol* **14**: 821–828

*© Cancer Research Campaign 1998*

Slevin ML, Clark PI, Joel SP, Malik S, Osborne RJ, Gregory WM, Lowe DG, Reznek RH and Wrigley PFM (1989) A randomized trial to evaluate the effect of schedule on the activity of etoposide in small-cell lung cancer. *J Clin Oncol* **7**: 1333–1340

Smit EF, Carney DN, Harford P, Sleijfer DT and Postmus PE (1989) A phase II study of oral etoposide in elderly patients with small cell lung cancer. *Thorax* **44**: 631–633

Smith IE, Harland SJ, Robinson BA, Evans BD, Goodhart LC, Calvert AH, Yarnold J, Glees JP, Baker J and Ford HT (1985) Carboplatin: a very active new cisplatin analog in the treatment of small cell lung cancer. *Cancer Treat Rep* **69**: 43–46

Smith IE, Evans BD, Gore ME, Vincent MD, Repetto L, Yarnold JR and Ford HT (1987) Carboplatin (paraplatin; JM8) and etoposide (VP-16) as first-line combination therapy for small-cell lung cancer. *J Clin Oncol* **5**: 185–189

Souhami RL, Spiro SG, Rudd RM, de Elvira M-CR, James LE, Gower NH, Lamont A and Harper PG (1997) Five-day oral etoposide treatment for advanced small-cell lung cancer: randomized comparison with intravenous chemotherapy. *J Natl Cancer Inst* **89**: 577–580

Wolf M, Tessen H-W, Goerg C, Achterrath W, Drings P and Havemann K (1991) Determining carboplatin/etoposide dosage in extensive stage small-cell lung cancer (SCLC). *Ann Oncol* **2**: 361–364

World Health Organization (1979) *WHO Handbook for Reporting Results of Cancer Treatment*, Vol. 48. WHO Offset Publication. World Health Organization: Geneva

© Cancer Research Campaign 1998